# Supplementary material for: Exploring the Critical Driving Forces and Strategy Adoption Paths of Professional Competency Development for Various Emergency Physicians Based on the Hybrid MCDM Approach
Source: Healthcare (Basel). 2023 Feb 6;11(4):471. doi: 10.3390/healthcare11040471 (PMC9957007; doi:10.3390/healthcare11040471)
Supplement: Supplementary file 1 [file healthcare-11-00471-s001.zip › healthcare-2050554-supplementary.pdf]

healthcare

MDPI

Supplementary File

1. The DEMATEL (Decision Making Trial and Evaluation Laboratory) approach

(1) Find the original average matrix

Through the online questionnaires survey, one hundred seventeen participants respond to the influence effect between the aspects by their experience from 0-4, where “0” means “no influence effect between the aspects" and "4" means “the highest effect between aspects." Subsequently, "1," "2," and "3" mean "low influence effect," "moderate influence effect," and "high influence effect," respectively. The effect of the PK (professional knowledge) aspect on the aspect of PS (professional skills) is 3.419, which indicates a high influence effect. The effect of the aspect of PS (professional skills) on the aspect of PL (professional literacy) is 2.521, which indicates a "medium influence effect," as demonstrated in Table S1.

Table S1. The original influence matrix.

Aspects	PK	PS	PL	CS	Total
Professional competence (PK)	0.000	**3.419**	2.547	2.735	8.701
Professional skills (PS)	3.282	0.000	**2.521**	2.581	8.385
Professional literacy (PL)	2.812	2.872	0.000	3.000	8.684
Care services (CS)	2.701	2.607	2.889	0.000	8.197
Total	8.795	8.897	7.957	8.316	-

(2) Estimate the direct influence matrix ($\boldsymbol{D}$)

The direct influence matrix ($\boldsymbol{D}$) was obtained from the “original influence matrix (A)” introduced by Equations (1) and (2), as shown in Table S2. In the direct influence matrix, the numerical value of diagonal items is all 0, and the sum of a row is at most equal to 1, as illustrated in Table S3. The overall direct influence can be obtained by calculating the columns and rows sum. The sum of the rows and columns for the **PK (professional competence)** was **1.966**, which is the most critical influence aspect. On the other hand, the sum of the rows and columns for **CS (care services)** was **1.856**, which is the least essential influence aspect, as shown in **Table S4**.

$$\boldsymbol{D} = s\boldsymbol{A}, \quad s > 0 \tag{1}$$

where

$$s = \min_{i,j} [1/\max_{1\le i\le n} \sum_{j=1}^{n} a_{ij}, 1/\max_{1\le j\le n} \sum_{i=1}^{n} a_{ij}], \quad i, j = 1, 2, ..., n \tag{2}$$

and $\lim_{m\to\infty} \boldsymbol{D}^m = [0]_{n\times n}$, where $\boldsymbol{D} = [x_{ij}]_{n\times n}$,

when $0 < \sum_{j=1}^{n} x_{ij}, \sum_{i=1}^{n} x_{ij} \le 1$ at least one $\sum_{j=1}^{n} x_{ij}$ or $\sum_{i=1}^{n} x_{ij}$ equal one, and only one row sum or column sum equal one. So, we can guarantee $\lim_{m\to\infty} \boldsymbol{D}^{m-1} = [0]_{n\times n}$.

Table S2. The direct influence matrix $(\boldsymbol{D})$.

Aspects	PK	PS	PL	CS	Total
Professional competence (PK)	0.000	0.384	0.286	0.307	0.978
Professional skills (PS)	0.369	0.000	0.283	0.290	0.942
Professional literacy (PL)	0.316	0.323	0.000	0.337	0.976
Care services (CS)	0.304	0.293	0.325	0.000	0.921

Total	0.988	1.000	0.894	0.935	-

Table S3. The comparison analysis of the direct influence matrix $(\boldsymbol{D})$.

	Sum of row	Sum of column	Sum of row and column	Importance of influence
Professional competence (PK)	0.978	0.988	**1.966**	1
Professional skills (PS)	0.942	1.000	1.942	2
Professional literacy (PL)	0.976	0.894	1.870	3
Care services (CS)	0.921	0.935	**1.856**	4

(3) Generate the indirect influence matrix

Derived from Equation (3), we can obtain the indirect influence matrix (***ID***), as demonstrated in **Table S4.**

$$\boldsymbol{ID} = \sum_{i=2}^{\infty} \boldsymbol{D}^i = \boldsymbol{D}^2(\boldsymbol{I}-\boldsymbol{D})^{-1} \quad (3)$$

Table S4. The indirect influence matrix ($\boldsymbol{ID}$).

Aspects	PK	PS	PL	CS	Total
Professional competence (PK)	5.265	5.203	4.795	4.949	20.211
Professional skills (PS)	5.029	5.171	4.670	4.826	19.696
Professional literacy (PL)	5.184	5.225	4.856	4.934	20.200
Care services (CS)	4.970	5.017	4.574	4.810	19.370
Total	20.447	20.616	18.895	19.519	-

(4) Evaluate the full influence matrix (***T***)

The ***T*** (full influence matrix) can be derived through Equations (4) or (5). The ***T*** includes multiple items shown as Equation (6), and T (full influence matrix) illustrated in **Table S5**. $\{d_i\}$ is the sum vector of the row value. $\{r_j\}$ is the sum vector of the column value. We then let $i = j$, the sum vector of the row value plus the column value, $\{d_i + r_i\}$ indicate the ***T*** (full influence matrix). The aspect relationship is stronger as the value of $\{d_i + r_i\}$ is higher. In contrast to $\{d_i + r_i\}$, the value of $\{d_i - r_i\}$ means the net influence relationship. If $d_i - r_i > 0$, it means the degree of influencing others is stronger than the degree of being influenced; otherwise, $d_i - r_i < 0$.

$$\boldsymbol{T} = \boldsymbol{D} + \boldsymbol{ID} = \sum_{i=1}^{\infty} \boldsymbol{D}^i \quad (4)$$

$$\boldsymbol{T} = \sum_{i=1}^{\infty} \boldsymbol{D}^i = \boldsymbol{D}(\boldsymbol{I}-\boldsymbol{D})^{-1} \quad (5)$$

$$\boldsymbol{T} = [t_{ij}], \quad i, j \in \{1, 2, \ldots, n\} \quad (6)$$

$$\boldsymbol{d} = \boldsymbol{d}_{n\times 1} = [\sum_{j=1}^{n} t_{ij}]_{n\times 1} = (d_1, \ldots, d_i, \ldots, d_n) \quad (7)$$

$$\boldsymbol{r} = \boldsymbol{r}_{n\times 1} = [\sum_{i=1}^{n} t_{ij}]'_{1\times n} = (r_1, \ldots, r_j, \ldots, r_n) \quad (8)$$

Table S5. The full influence matrix (T).

Aspects	PK	PS	PL	CS	Total
Professional knowledge (PK)	5.265	5.587	5.081	5.256	21.188

Professional skills (PS)	5.398	5.171	4.953	5.116	20.638
Professional literacy (PL)	5.500	5.548	4.856	5.271	21.176
Care services (CS)	5.274	5.310	4.899	4.810	20.292
Total	21.436	21.616	19.789	20.453	-

As illustrated in **Table S6**, the PK aspect has the highest degree of full influence (d_1+r_1=42.624), and the CS aspect has the lowest degree of full influence (d_4+r_4=40.745). The PL aspect (d_3-r_3= 1.387) also has the highest level of net influence. The PS aspect (d_2-r_2=-0.978) has the lowest degree of net influence. The other net influence order is as follows: PK aspect (d_1-r_1= – 0.248) and CS aspect (d_4-r_4= – 0.160).

Table S6. The degree of full influence.

Aspects	$\{d_i\}$	$\{r_i\}$	$\{d_i+r_i\}$	$\{d_i-r_i\}$
Professional knowledge (PK)	21.188	21.436	**42.624**	-0.248
Professional skills (PS)	20.638	21.616	42.254	**-0.978**
Professional literacy (PL)	21.176	19.789	40.965	**1.387**
Care services (CS)	20.292	20.453	**40.745**	-0.160

(5) Obtain the network relation map (NRM)

The study chooses one of the strictly triangular matrices, and the matrix's diagonal items are all 0. The matrix contains a strictly lower and strictly upper triangular matrix. In addition, while the strictly lower triangular matrix and the strictly upper are the same, their symbols are opposite. The full influence matrix can be obtained by Equation (4) and Equation (5), as shown in **Table S5**. The net influence matrix (***Tnet***) can be produced by Equation (9), as shown in **Table S7**. The X and Y values can be acquired through the ($d+r$) value and ($d-r$) value, as shown in **Table S6**. The NRM (network relation map) can be drawn, as illustrated in **Figure 3 and Table S7**.

$$\boldsymbol{T_{net}} = [t_{ij} - t_{ji}], \qquad i, j \in \{1, 2, ..., n\} \tag{9}$$

The aspects of PK, PL, and CS are the influencing aspects, and the aspect of PS is the affected aspect. The DEMATEL-NRM can calculate the degree of influence of each aspect and obtain the net influence relation among these four aspects. The PL aspect has a net influence on PS, PK, and CS. Besides, the CS aspect has a net influence on the PK and PS. The PK aspect has a net influence on PS. So, the PL aspect can enhance first, followed by the CS and PK aspects. The aspect of PS is the least essential improvement item among all aspects, as shown in **Table S7** and **Figure 3**.

Table S7. The net influence matrix (***Tnet***). .

Aspects	PK	PS	PL	CS
Professional knowledge (PK)	-			
Professional skills (PS)	-0.189	-		
Professional literacy (PL)	0.420	0.595	-	
Care services (CS)	0.018	0.194	-0.372	-

2. The PCA (Principal component analysis) approach

Principal components analysis (PCA) is a technique for analyzing and simplifying large data sets. The original variables are used to synthesize new variables to achieve the purpose of variable reduction and preserve the critical information provided by the data sets. One component can extract PKP1 (diagnostic studies & pharmacotherapy, PKP1) and the square sum (79.993%). Diagnostic studies (PK1), pharmacotherapy (PK2), differential

diagnosis (PK3), and observational reassessment (PK4) can combine into the first principal component, PKP1 (diagnostic studies & pharmacotherapy), shown in **Table S8**.

Table S8. The PCA analysis of PK (professional knowledge) aspect.

			Components	
Aspects	Components	Criteria	1	Community
Professional knowledge (PK)	Diagnostic studies & Pharmacotherapy (PKP1)	Diagnostic studies (PK1)	0.915	0.838
		Pharmacotherapy (PK2)	0.911	0.830
		Differential diagnosis (PK3)	0.897	0.805
		Observational reassessment (PK4)	0.852	0.726
	Eigenvalue λ	3.200		
	% of Variance	79.993		
	Cumulative (%)	79.993		
	Cronbach's α	0.916		

3. The ANP (Analytic network procedure) approach

(1). Determine the research problem and build the evaluation of the framework

Complex decision-making can be simplified by utilizing the relation structure of the evaluation system. Researchers should determine all possible aspects/criteria to establish the evaluation structure through the literature review and expert discussion. In the evaluation system, the aspects influence each other. The study calculates the relation weights of aspects with the ANP technique based on the NRM approach.

(2). The evaluation framework designs and questionnaires investigation

After determining the evaluation framework, the expert can understand the outer and inner dependence influences between the aspects. Hence, the study evaluates the relative importance of the questionnaire survey.

(3). Establish the paired comparison matrices to evaluate the aspects/criteria' weights under consideration of dependence and feedback. The weights obtained by the ANP technique are as follows:

(1) Determine the aspects' relative importance paired comparison and obtain an $n \times n$ pairwise comparison matrix, where n means the number of components.

(2) Compute the logical judgment consistency by both the consistency index ($C.I.$) and the consistency ratio ($C.R.$). In general, $C.I.$ and $C.R.$ should be less than 0.1.

(4). Calculate the transposed and normalized full influence matrix

The full influence matrix (T) can be derived from Equation (4) or Equation (5). The d_i can be calculated by Equation (10) through the sum of the column of the full influence matrix (T). Then, the normalized full influence matrix (T_N) can be obtained through Equation (11), and the transposed and normalized full influence matrix (T_N^t) can be derived through Equation (12). T_N^t is the transposed-normalized full influence matrix, as shown in Table S9.

$$T=\begin{bmatrix} t_{11} & \cdots & t_{1j} & \cdots & t_{1n} \\ \vdots & \ddots & \vdots & \ddots & \vdots \\ t_{i1} & \cdots & t_{ij} & \cdots & t_{in} \\ \vdots & \ddots & \vdots & \ddots & \vdots \\ t_{n1} & \cdots & t_{nj} & \cdots & t_{nn} \end{bmatrix} \begin{matrix} \rightarrow d_1 = \sum_{j=1}^{n} t_{1j} \\ \\ \rightarrow d_i = \sum_{j=1}^{n} t_{ij} \\ \\ \rightarrow d_n = \sum_{j=1}^{n} t_{nj} \end{matrix} \quad (10)$$

where $d_i=\sum_{j=1}^{n} t_{ij}, i=1,2,\ldots,n$

$$T_N=\begin{bmatrix} t_{11}/d_1 & \cdots & t_{1j}/d_1 & \cdots & t_{1n}/d_1 \\ \vdots & \ddots & \vdots & \ddots & \vdots \\ t_{i1}/d_i & \cdots & t_{ij}/d_i & \cdots & t_{in}/d_i \\ \vdots & \ddots & \vdots & \ddots & \vdots \\ t_{n1}/d_n & \cdots & t_{nj}/d_n & \cdots & t_{nn}/d_n \end{bmatrix}=\begin{bmatrix} t_{11}^N & \cdots & t_{1j}^N & \cdots & t_{1n}^N \\ \vdots & \ddots & \vdots & \ddots & \vdots \\ t_{i1}^N & \cdots & t_{ij}^N & \cdots & t_{in}^N \\ \vdots & \ddots & \vdots & \ddots & \vdots \\ t_{n1}^N & \cdots & t_{nj}^N & \cdots & t_{nn}^N \end{bmatrix} \quad (11)$$

$$T_N^t=(T_N)'=\begin{bmatrix} t_{11}^N & \cdots & t_{i1}^N & \cdots & t_{n1}^N \\ \vdots & \ddots & \vdots & \ddots & \vdots \\ t_{1j}^N & \cdots & t_{ji}^N & \cdots & t_{nj}^N \\ \vdots & \ddots & \vdots & \ddots & \vdots \\ t_{1n}^N & \cdots & t_{in}^N & \cdots & t_{nn}^N \end{bmatrix} \quad (12)$$

Table S9. The transposed-normalized full influence matrix (T_N^t).

Aspects	PK	PS	SL	CS
Professional knowledge (PK)	0.248	0.262	0.260	0.260
Professional skills (PS)	0.264	0.251	0.262	0.262
Professional literacy (PL)	0.240	0.240	0.229	0.241
Care services (CS)	0.248	0.248	0.249	0.237
Total	1.000	1.000	1.000	1.000

(5). Calculate the weighted supermatrix (W_L)

The W_p (un-weighted supermatrix) is demonstrated in Equation (13), whereas the W_p is composed of many sub-matrices (W_{ij}). The researcher solves the relationship of dependency and feedback in the NRM (network relation map), and the ANP technique analyzes the sub-matrix weight by the paired comparison matrix, as displayed in Equation (14). If only a single aspect of the component exists, the sub-matrix is the unit matrix (*I*). When the aspect includes more than one component, the sum of the component weight equals one. As illustrated in Table S9, the W_L (weighted supermatrix) can be calculated by multiplying the T_N^t (transposed-normalized full influence matrix) and the W_p, or it can be derived through Equation (15). Therefore, when there is more than one component in each aspect, the W_L (weighted supermatrix) can be modified through Equation (15) and Equation (16), as illustrated in Tables S10-11. The supermatrix can gain through $(W_L \times W_L)^{2p+1}$ where p is determined by assumption. The ANP approach could calculate the weight of components and the reduced criteria is derived from the independent component obtained. The criteria weights can be constructed through the ANP approach. In the limitation process, multiples of the supermatrices *M* for 45 squares and the component weights can be acquired, as illustrated in Table S12.

$$W_P = \begin{bmatrix} W_{11} & \cdots & W_{1j} & \cdots & W_{1m} \\ \vdots & \ddots & \vdots & \ddots & \vdots \\ W_{i1} & \cdots & W_{ij} & \cdots & W_{im} \\ \vdots & \ddots & \vdots & \ddots & \vdots \\ W_{m1} & \cdots & W_{mj} & \cdots & W_{mm} \end{bmatrix} \quad (13)$$

$$W_{ij} = \begin{bmatrix} w_{P_{11}} & \cdots & w_{P_{1j}} & \cdots & w_{P_{1m}} \\ \vdots & & \vdots & & \vdots \\ w_{P_{i1}} & \cdots & w_{P_{ij}} & \cdots & w_{P_{im}} \\ \vdots & & \vdots & & \vdots \\ w_{P_{m1}} & \cdots & w_{P_{mj}} & \cdots & w_{P_{mm}} \end{bmatrix} = 1 \quad i = 1, 2, \ldots.m \quad j = 1, 2, \ldots\ldots m \quad (14)$$

$$\text{here } \sum_{i=1}^{m} w_{P_{i1}} = \sum_{i=1}^{m} w_{P_{ij}} = \sum_{i=1}^{m} w_{P_{im}} = 1$$

$$W_L = T_N^t \times W_P = \begin{bmatrix} t_{11}^N \times W_{11} & \cdots & t_{i1}^N \times W_{1j} & \cdots & t_{n1}^N \times W_{1m} \\ \vdots & & \vdots & & \vdots \\ t_{1j}^N \times W_{i1} & \cdots & t_{ji}^N \times W_{ij} & \cdots & t_{nj}^N \times W_{im} \\ \vdots & & \vdots & & \vdots \\ t_{1n}^N \times W_{m1} & \cdots & t_{in}^N \times W_{mj} & \cdots & t_{nn}^N \times W_{mm} \end{bmatrix} \quad (15)$$

$$t_{ji}^N \times W_{ij} = \begin{bmatrix} t_{11}^N \times w_{P_{11}} & \cdots & t_{i1}^N \times w_{P_{1j}} & \cdots & t_{n1}^N \times w_{P_{1m}} \\ \vdots & & \vdots & & \vdots \\ t_{1j}^N \times w_{P_{i1}} & \cdots & t_{ji}^N \times w_{P_{ij}} & \cdots & t_{nj}^N \times w_{P_{im}} \\ \vdots & & \vdots & & \vdots \\ t_{1n}^N \times w_{P_{m1}} & \cdots & t_{in}^N \times w_{P_{mj}} & \cdots & t_{nn}^N \times w_{P_{mm}} \end{bmatrix} \quad (16)$$

Table S10. Un-weighted supermatrix (W_p).

Aspects	Components	PKP1	PSP1	PLP1	CSP1
Professional knowledge (PK)	Diagnostic studies & pharmacotherapy (PKP1)	1.000	1.000	1.000	1.000
Professional skills (PS)	Emergency stabilization & management (PSP1)	1.000	1.000	1.000	1.000
Professional literacy (PL)	Professional ethics & communication (PLP1)	1.000	1.000	1.000	1.000
Care services (CS)	Care management & teamwork (CSP1)	1.000	1.000	1.000	1.000
	Total	4.000	4.000	4.000	4.000

Table S11. Weighted supermatrix (W_L).

Aspects	Components	PKP1	PSP1	PLP1	CSP1
Professional knowledge (PK)	Diagnostic studies & pharmacotherapy (PKP1)	0.257	0.257	0.257	0.257
Professional skills (PS)	Emergency stabilization & management (PSP1)	0.259	0.259	0.259	0.259
Professional literacy (PL)	Professional ethics & communication (PLP1)	0.238	0.238	0.238	0.238
Care services (CS)	Care management & teamwork (CSP1)	0.246	0.246	0.246	0.246
	Total	1.000	1.000	1.000	1.000

Table S12. Limited supermatrix.

Aspects	Components	PKP1	PSP1	PLP1	CSP1
Professional knowledge (PK)	Diagnostic studies & pharmacotherapy (PKP1)	0.257	0.257	0.257	0.257
Professional skills (PS)	Emergency stabilization & management (PSP1)	0.259	0.259	0.259	0.259
Professional literacy (PL)	Professional ethics & communication (PLP1)	0.238	0.238	0.238	0.238
Care services (CS)	Care management & teamwork (CSP1)	0.246	0.246	0.246	0.246
	Total	1.000	1.000	1.000	1.000

(6). Compute the component weights

As illustrated in Table 9 (in the original article), the parenthetic value means the weights of the aspects/components.

4. The VIKOR (Vlse kriterijumska Optimizacija I Kompromisno Resenje)

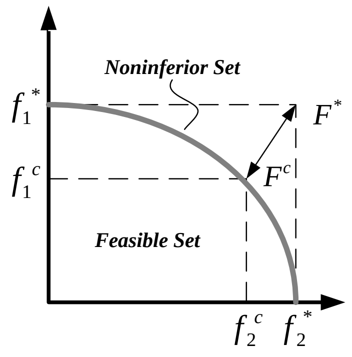

Figure S1. Ideal and compromise solutions.

As illustrated in Figure S1, where: F^* is the ideal solution. f_1^* represents the ideal value (also called the aspired/desired level) of Factor 1. f_2^* represents the ideal value (the aspired/desired level) of Factor 2. The compromise solution, F^c, is a feasible solution that is "closest" to the ideal F^*. A compromise means an agreement established by mutual concessions. The VIKOR approach is presented with the following steps:

Step 1: Determines the best f_k^* value and the worst f_k^- value in aspect/component i.

$$f_k^* = \left\{\left(\max_k f_{ik} \mid k \in I_1\right), \left(\min_k f_{ik} \mid k \in I_2\right); \text{or setting the aspired level for } i \text{ criterion}\right\}, \forall k \quad (17)$$

$$f_k^- = \left\{\left(\min_k f_{ik} \mid k \in I_1\right), \left(\max_k f_{ik} \mid k \in I_2\right); \text{or setting the worst level for } i \text{ criterion}\right\}, \forall k \quad (18)$$

where: i is the criterion; k is the kth alternative; f_{ik} is the performance value of the ith criterion of kth alternative; I_1 is the cluster of utility-oriented criteria; I_2 is the cluster of the cost-oriented criteria; f_i^* is the positive-ideal solution; and f_i^- is the positive-ideal solution.

Step 2: Evaluates the values S_k and Q_k, $k = 1,2,.....,m$, using the relationships.

Let r_{ik} be $r_{ik} = (|f_i^* - f_{ik}|)/(|f_i^* - f_i^-|)$.

$$d_k^p = \left\{\sum_{i=1}^{n}[w_i(|f_i^* - f_{ik}|)/(|f_i^* - f_i^-|)]^p\right\}^{1/p} = \left\{\sum_{i=1}^{n}[w_i r_{ik}]^p\right\}^{1/p}, \quad p \geq 1 \quad (19)$$

$$S_k = d_k^{p=1} = \sum_{i=1}^{n} w_i r_{ik}, \qquad \sum_{i=1}^{n} w_i = 1 \tag{20}$$

$$Q_k = d_k^{p=\infty} = \max_k \{ r_{ik} \mid i = 1, 2, \ldots, n \}, \tag{21}$$

where S_k shows the average gap for achieving the aspired/desired level; Q_k illustrates the maximal degree of regret for prior improvement of the gap aspect/component. w_i is the weight of aspect/component i and $i = 1, 2, \ldots, n$, expressing the relative importance value of the criteria gained via the application of the ANP approach.

Step 3: Computes the index values R_k, $k = 1, 2, \ldots, m$, using the relationship:

$$R_k = v(S_k - S^*)/(S^- - S^*) + (1 - v)(Q_k - Q^*)/(Q^- - Q^*) \tag{22}$$

$$S^* = \min_k S_k, \quad S^- = \max_k S_k$$

$$Q^* = \min_k Q_k, \quad Q^- = \max_k Q_k$$

where $S^* = \min_k S_k$ (showing the minimal average gap is the best, but we also can set $S^* = 0$), $S^- = \max_k S_k$ (we can set $S^- = 1$); $Q^* = \min_k Q_k$ (illustrating the minimal degree of regret is the best, but we also can set $Q^* = 0$), $Q^- = \max_k Q_k$ (we can set $Q^- = 1$). We also can re-write Equation (15), $R_k = vS_k + (1 - v)Q_k$.

Step 4: Rank the alternatives

When $0 \le v \le 1$ and when $v > 0.5$, this indicates S is emphasized more than Q in Equation (22), whereas when $v < 0.5$, this indicates Q is emphasized more than S in Equation (22). More specifically, when $v = 1$, it represents an alternative evaluation process that could use the strategy of maximum group utility. Whereas when $v = 0$, it represents an alternative evaluation process that could adopt the strategy of minimum individual regret, which is obtained among the maximum individual regrets/gaps of lower-level dimensions of each project (or aspects/objectives). The weight (v) would affect the ranking order of the aspects/components, and the decision-makers usually determine it. R_k is applied to determine the CDI. R_k could also consider the index of the maximum group utility and the minimum individual regret of the "opponent," where a smaller R_k is better and $0 \leqq R_k \leqq 1$. Competency evaluation index

5. The result of the VIKOR approach

(1). Determines the best f_i^* value and the worst f_i^- value in aspect/component *i*.

In Equation (17) and Equation (18), as illustrated in **Table S13**, k is the kth alternative of factor i; f_{ik} is the performance value of the aspects/criteria i in alternative k; f_i^* is the positive-ideal solution (setting the desired/aspired level by decision-making from customers' needs); and f_i^- is the negative-ideal solution (setting the worst value by decision-making from users). f_i^* is assumed to be 10 and f_i^- is assumed to be 0. This result can aid decision-makers in improving the satisfaction gap.

Table S13. The score of f_{ik}.

Aspects	Weight	PGYs (Staff A)	Residents (Staff B)	Visiting staffs (Staff C)	f_{vk}^*	f_{vk}^-
Professional knowledge (PK)	0.257	8.426	8.215	8.568	10	0

Professional skills (PS)	0.259	8.051	8.299	8.257	10	0
Professional literacy (PL)	0.238	8.000	7.750	8.297	10	0
Care services (CS)	0.246	7.955	7.535	8.014	10	0

(2). Computes the values S_{vk} and Q_{vk}, $k=1,2,...,m$, through the relationships weight.

In referring to Equation (20) and Equation (21), w_i are the component weights, expressing the relative importance value of the components via the ANP approach. The lowest S_{vk} is 0.171 in Staff C and the highest S_{vk} is 0.204 in Staff B as illustrated in **Table S14**. In addition, the lowest Q_{vk} is 0.199 in Staff C and the highest Q_{vk} is 0.247 in Staff B among the CDI.

Table S14. The weighted value of the components of f_{vk}.

Aspects	Weight	PGYs (Staff A)	Residents (Staff B)	Visiting staffs (Staff C)
Professional knowledge (PK)	0.257	0.157	0.178	0.143
Professional skills (PS)	0.259	0.195	0.170	0.174
Professional literacy (PL)	0.238	0.200	0.225	0.170
Care services (CS)	0.246	0.205	0.247	0.199
S_{vk}		0.189	0.204	0.171
Q_{vk}		0.205	0.247	0.199

(3). Computes the index values R_{vk}, $k=1,2,...,m$, using the relationship:

In referring to Equation (22), $\min_k S_{vk}$ is with a maximum group utility ("majority" rule) and $\min_k Q_{vk}$ is with a minimum individual regret of the "opponent." R_{vk} is the indicator of the in alternative k (the smaller is the better). The R_{vk} would reduce as v rises from 0 to 1, as shown in **Table S15**.

Table S15. R_{vk} under different v for CDI (competency development indicators).

v	PGYs	Residents	Visiting staffs
	(Staff A)	(Staff B)	(Staff C)
0.00	0.205	0.247	0.199
0.10	0.203	0.242	0.196
0.20	0.201	0.238	0.193
0.30	0.200	0.234	0.190
0.40	0.198	0.230	0.188
0.50	0.197	0.225	0.185
0.60	0.195	0.221	0.182
0.70	0.194	0.217	0.180
0.80	0.192	0.213	0.177
0.90	0.190	0.208	0.174
1.00	0.189	0.204	0.171

(4). Rank the alternatives

In **Table S16**, the R_{vk} under different v illustrated and R_{vk} (here v =0.5) can apply to evaluate the CDI. The R_{vk} can also evaluate the index of the minimum individual regret and the maximum group utility of the "opponent," where R_{vk} means smaller is better and $0 \leqq R_{vk} \leqq 1$, The researcher adopts $1-R_{vk}$ for the system evaluation, which means $1-R_{vk}$;

bigger is better. When the v value of CDI is 0.5, then V= R_{vk} and CDI=1− R_{vk} . Therefore, the CDI of different alternatives can be obtained. Under v=0.0, the lowest CDI is 0.753 belonging to Staff B, the highest CDI is 0.801 belonging to Staff C. In the ranking of the CDI of three staffs of emergency physicians, Staff C is better than other staffs (PGYs, and Residents), as illustrated in **Table S16**.

Table S16. The CDI (competency development indicators) (1- R_{vk}) under different v.

v	PGYs (Staff A)	Residents (Staff B)	Visiting staffs (Staff C)
0.00	0.795	0.753	0.801
0.10	0.797	0.758	0.804
0.20	0.799	0.762	0.807
0.30	0.800	0.766	0.810
0.40	0.802	0.770	0.812
0.50	0.803	0.775	0.815
0.60	0.805	0.779	0.818
0.70	0.806	0.783	0.820
0.80	0.808	0.787	0.823
0.90	0.810	0.792	0.826
1.00	0.811	0.796	0.829
